# Point-of-Care Ultrasonography in Advanced Nephrology Nursing Practice: Seeing Beyond the Numbers

**DOI:** 10.3390/diagnostics15243196

**Published:** 2025-12-14

**Authors:** Antoni Garcia-Lahosa, Sergio Moreno-Millán, Maria Cruz Sanchez-García, Miguel Sanchez-Cardenas, Christiane Steiss, Wilmer Jim Escobar, Miguel Nuñez-Moral, Jordi Soler-Majoral, Fredzzia Graterol Torres, Jordi Ara, Jordi Bover, J. Emilio Sánchez-Alvarez, Faeq Husain-Syed, Abhilash Koratala, Gregorio Romero-González, Sonia Fernández-Delgado, Nestor Rodríguez-Chitiva, Elisabeth Marcos-Ballesteros

**Affiliations:** 1 Nursing Team, Nephrology Department, University Hospital Germans Trias I Pujol, Carretera de Canyet s/n, 08916 Badalona, Spain; agarciala.germanstrias@gencat.cat (A.G.-L.); smorenom.germanstrias@gencat.cat (S.M.-M.); mcsanchez.germanstrias@gencat.cat (M.C.S.-G.); emarcos.germanstrias@gencat.cat (E.M.-B.); 2 Faculty of Nursing, Universidad El Bosque, Bogotá 110131, Colombia; sanchezcmiguel@unbosque.edu.co; 3 PHV Dialysis Center, University Hospital Giessen and Marburg, 35392 Giessen, Germany; steiss.christiane@phv-dialyse.de; 4 Asociación Vascular de Enfermería de Perú (AVEDPERU), 153116 Lima, Peru; jimccv@gmail.com; 5 Latin American Association of Nephrology Nursing (ALAEN), Montevideo 11300, Uruguay; 6 Peritoneal Dialysis Unit, University Hospital Central of Asturias (HUCA), 33011 Oviedo, Spain; miguel.nunez@sespa.es; 7 Faculty of Nursing, University of Oviedo, 33003 Oviedo, Spain; 8 Nephrology Department, University Hospital Germans Trias I Pujol, 08916 Badalona, Spain; jsoler.germanstrias@gencat.cat (J.S.-M.); fgrateroltorres@bwh.harvard.edu (F.G.T.); jordiaradelrey.germanstrias@gencat.cat (J.A.); jbover.ics@gencat.cat (J.B.); nyrodriguezc.germanstrias@gencat.cat (N.R.-C.); 9 REMAR-IGTP Group, Germans Trias i Pujol Research Institute, Can Ruti Campus, 08916 Badalona, Spain; 10 Nephrology Department, University Hospital Central of Asturias (HUCA), 33011 Oviedo, Spain; joseemilio.sanchez@sespa.es; 11 Department of Internal Medicine II, University Hospital Giessen and Marburg, Justus-Liebig-University Giessen, 35392 Giessen, Germany; faeq.husain-syed@innere.med.uni-giessen.de; 12 International Renal Research Institute of Vicenza, 36100 Vicenza, Italy; 13 Division of Nephrology, Medical College of Wisconsin, Milwaukee, WI 53226, USA; akoratala@mcw.edu; 14 Hemodialysis and Home Therapies Unit, University Hospital Germans Trias I Pujol, 08916 Badalona, Spain; sfernandezd.germanstrias@gencat.cat

**Keywords:** advanced nursing practice, congestion assessment, nephrology nursing, point-of-care ultrasonography, vascular access management

## Abstract

Chronic kidney disease (CKD) affects nearly 850 million people worldwide, and most patients with kidney failure are treated with kidney replacement therapy. Despite technological progress, venous congestion remains a major determinant of morbidity and mortality, and is often underdetected by conventional tools such as clinical evaluation, weight changes, blood pressure measurement, or bioimpedance. Point-of-care ultrasonography (PoCUS) has transformed this diagnostic landscape by providing real-time, physiology-based insights into both left- and right-sided filling pressures. In dialysis care, multiple or confluent B-lines and subtle pleural irregularities suggest elevated pulmonary capillary wedge pressure, while a dilated inferior vena cava (IVC) with reduced collapsibility and increased portal vein pulsatility indicate elevated right atrial pressures. Integrating these sonographic findings into a multiparametric assessment that also includes clinical assessment, bioimpedance, and biosensor feedback enhances diagnostic sensitivity and refines fluid management. Advanced practice nurses (APNs) trained in PoCUS can perform focused examinations of the lungs, IVC, portal venous system, arteriovenous access, and skeletal muscle, translating ultrasound findings into physiological interpretations that guide individualized ultrafiltration strategies and patient care. Nutritional ultrasound (NUS) further complements congestion assessment by quantifying muscle mass and quality, linking nutritional reserve and functional status with hemodynamic tolerance. The implementation of structured education, competency-based training, and standardized scanning protocols allows nurses to incorporate these techniques safely and reproducibly into daily dialysis workflows. By integrating PoCUS and NUS within interdisciplinary decision-making, nursing practice evolves from procedural to diagnostic, supporting early identification of congestion, protection of vascular access, and detection of malnutrition. This multiparametric, physiology-guided approach exemplifies the concept of precision nursing, where patient evaluation becomes continuous, individualized, and grounded in real-time pathophysiological insight.

## 1. Introduction

Chronic kidney disease (CKD) affects nearly 850 million people worldwide and represents a major global health burden [1,2]. Most patients with kidney failure requiring kidney replacement therapy are treated with dialysis, with kidney transplantation accounting for a much smaller proportion. According to the International Society of Nephrology (ISN) Global Kidney Health Atlas 2023, approximately 65–70% of treated patients receive hemodialysis (HD) and 20–25% undergo peritoneal dialysis (PD), while fewer than 15% live with a functioning kidney transplant [3]. Despite technological advances, venous congestion continues to be the “silent enemy” of dialysis care, often unrecognized until late stages, when dyspnea, pitting edema, or hypotension appear [4,5,6]. Conventional tools such as interdialytic weight changes, blood pressure trends, or bioimpedance spectroscopy frequently fail to detect subclinical congestion or to differentiate between intravascular and interstitial overload [6,7,8].

Point-of-care ultrasonography (PoCUS) has expanded this diagnostic landscape by providing real-time, physiology-based insights into cardiovascular congestion [9]. Lung ultrasound findings such as multiple or confluent B-lines and mild pleural irregularities point toward elevated left-sided filling pressure (increased pulmonary capillary wedge pressure [PCWP]), whereas a dilated IVC with reduced inspiratory collapse reflects elevated right atrial pressure [10,11,12,13,14]. In addition to quantifying extravascular lung water, PoCUS can identify indirect sonographic markers that may indicate elevated cardiac filling pressures. Complementary Doppler-based indices, such as portal and hepatic venous flow patterns integrated into the Venous Excess Ultrasound (VExUS) score, further characterize systemic venous congestion and its dynamic evolution during ultrafiltration [15,16,17]. In hemodialysis populations, several studies have demonstrated that lung ultrasound and venous ultrasound markers provide diagnostic information not captured by clinical examination or weight-based assessment. Lung congestion quantified by B-lines has been associated with mortality and cardiovascular events in patients receiving maintenance hemodialysis [7,18], while dynamic changes in venous congestion patterns have been linked to ultrafiltration tolerance and hemodynamic instability [9]. In addition, randomized and observational evidence, including the LUST trial, has shown that ultrasound-guided strategies may improve the safety of decongestion by reducing intradialytic hypotension in selected high-risk patients [19]. Together, these findings support the integration of PoCUS as a complementary diagnostic tool in routine dialysis care. Within this evolving diagnostic landscape, the contribution of advanced practice nurses (APNs) has become increasingly relevant. APNs possess advanced clinical reasoning skills and expanded scope of practice that include focused assessment, protocol-driven decision-making, and integration of physiologic data at the bedside. As congestion assessment shifts toward a more physiology-based and imaging informed model, PoCUS offers a natural extension of these competencies by enabling nurses to obtain real-time hemodynamic information that complements traditional clinical evaluation. This contextual framework explains why APN-led PoCUS is gaining traction in nephrology practice and supports its introduction early in the dialysis diagnostic workflow.

**Figure 1:**
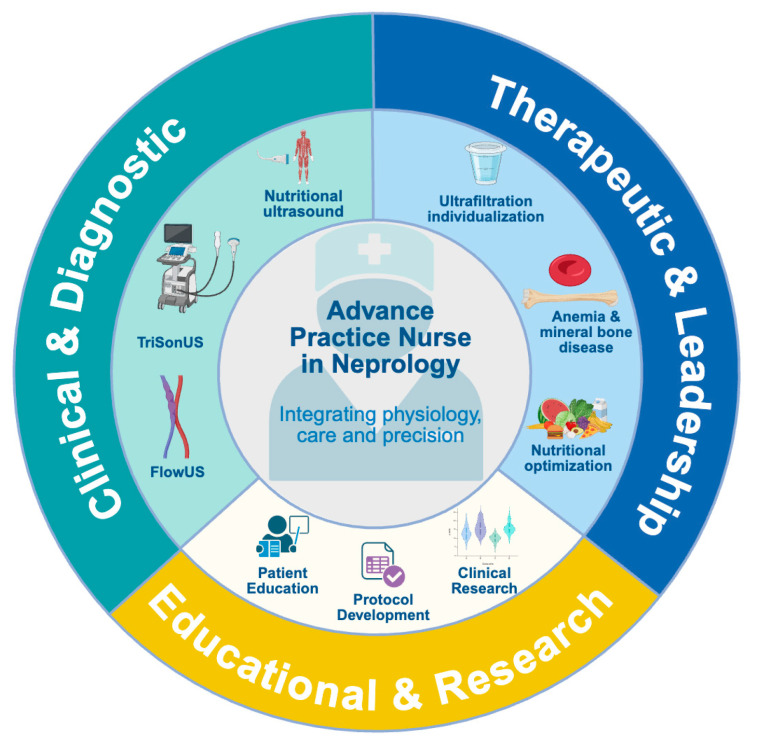
Core Competencies of Advanced Practice Nursing in Nephrology. Conceptual wheel depicting the main domains of advanced nursing practice. At the center, the Advanced Practice Nurse in Nephrology (APN-N) integrates physiology, care, and precision through three interconnected axes: (1) Clinical & Diagnostic Reasoning, including PoCUS applications such as TRISONUS (three-site sonography: LUS, IVC, and PV), FLOWUS (vascular ultrasound for access monitoring), and nutritional ultrasound; (2) Therapeutic & Leadership, encompassing ultrafiltration individualization, anemia and bone-mineral management, and nutritional optimization; (3) Educational & Research, including patient education, protocol development, and clinical research. This framework illustrates how PoCUS extends nursing competence from procedure to diagnostic reasoning within precision nephrology. Abbreviations: APN-N, advanced practice nurse in nephrology; PoCUS, point-of-care ultrasound; TRISONUS, three-site sonographic framework (lung, IVC, and PV); FLOWUS, vascular ultrasound assessment.

In recent years, diagnostic ultrasound has become an essential component of precision nephrology and is now used not only by physicians but also by advanced practice nurses (APNs) with appropriate training [20,21]. Within this evolving model, nephrology nurses integrate focused PoCUS examinations into routine HD care to evaluate congestion, assess vascular access, and support individualized clinical decision-making [20,22]. This approach aligns with the core competencies of advanced nursing practice, clinical reasoning, diagnostic assessment, and interdisciplinary collaboration, transforming the dialysis unit into a diagnostic environment where congestion can be visualized, interpreted, and managed proactively (Figure 1) [23]. In addition to hemodynamic profiling, ultrasound has also emerged as a tool to evaluate nutritional and functional status. Nutritional ultrasound (NUS) allows standardized bedside measurement of muscle thickness, cross-sectional area, and echogenicity, as well as preperitoneal and visceral fat. These parameters provide objective markers of protein-energy wasting and sarcopenia that are largely independent of hydration status [24,25]. Integrating NUS into dialysis care links structural and metabolic reserve with hemodynamic tolerance during ultrafiltration and complements congestion assessment in a clinically meaningful way.

The aim of this review is to summarize the diagnostic and educational value of PoCUS in advanced nephrology nursing practice, focusing on its role in estimating cardiac filling pressures, guiding fluid management, and strengthening diagnostic competence within multidisciplinary dialysis teams.

## 2. Clinical and Diagnostic Challenges in Volume Assessment

Accurate assessment of volume status remains one of the greatest challenges in the management of patients receiving hemodialysis (HD). Traditional approaches that rely on changes in body weight, blood pressure, or clinical examination often provide only indirect or delayed signs of hemodynamic overload [26]. The classic concept of “dry weight,” defined as the lowest post-dialysis weight tolerated without signs of hypo or hypervolemia, has guided ultrafiltration prescriptions for decades [4]. However, this approach is largely empirical and fails to reflect the complex relationship between vascular filling pressures, tissue fluid shifts, and cardiovascular tolerance to ultrafiltration.

Clinical signs such as pitting edema, crackles, or jugular venous distension appear only when congestion is already advanced, and their absence does not exclude elevated cardiac filling pressures [27,28]. Likewise, bioimpedance spectroscopy estimates extracellular water but cannot differentiate between interstitial and intravascular compartments, nor assess the dynamic refilling capacity during fluid removal [7,8,29]. As a result, many patients who appear “euvolemic” by clinical and bioimpedance criteria may still have significant pulmonary or venous congestion detectable only by hemodynamic imaging [30].

Recent studies have emphasized that congestion in dialysis patients is not merely a problem of “fluid excess” but rather a hemodynamic disorder characterized by elevated cardiac filling pressures and abnormal venous return [31,32]. This distinction is clinically relevant: vascular congestion, driven by increased venous pressures, may exist without overt edema, whereas tissue congestion results from interstitial accumulation that may persist even after apparent ultrafiltration success [9,33]. Understanding these phenotypes is essential to tailor fluid management and to avoid the circular pattern of under- and over-dehydration that drives cardiovascular instability in HD.

Taken together, these limitations highlight that no single tool can reliably characterize volume status in hemodialysis. Rather than replacing established methods, PoCUS provides complementary, physiology-based information that enhances clinical interpretation when integrated with symptoms, blood pressure trends, and bioimpedance findings.

## 3. A Multiparametric Ultrasound Approach to Assessing Congestion

### 3.1. Lung Ultrasound

Lung ultrasonography (LUS) is the most accessible and validated PoCUS application for detecting left-sided congestion [34,35,36]. Its diagnostic value lies in the identification of B-lines, which are vertical, laser-like artifacts arising from the pleural line indicating increased interstitial fluid and disruption of the normal alveolar–capillary interface [11,36,37]. Multiple or confluent B-lines are strongly associated with elevated pulmonary capillary wedge pressure and interstitial edema, including in patients who may appear clinically asymptomatic. Accordingly, LUS can reveal physiologically meaningful pulmonary congestion that may not be evident on routine clinical examination [18,30,38].

The LUST trial, conducted in patients on chronic hemodialysis with high cardiovascular risk, showed that a lung ultrasound-guided ultrafiltration strategy was safe and effectively reduced pulmonary congestion, and it significantly lowered the frequency of intradialytic hypotension compared with standard care. However, the intervention did not improve the primary or secondary endpoints, including mortality or major cardiovascular events, and its conclusions cannot be extrapolated to the general hemodialysis population [19]. When interpreted alongside broader evidence, these findings reinforce the importance of detecting subclinical pulmonary congestion. Although derived from acute heart failure populations rather than hemodialysis, the study by Rivas-Lasarte et al. demonstrated that approximately half of the patients discharged with residual subclinical congestion were readmitted within 15 days with overt decompensation, and that their prognosis was as poor as that of patients discharged with clinical congestion [30]. This concept that physiologically meaningful congestion may remain clinically silent yet strongly associated with adverse outcomes, has not been systematically explored in hemodialysis, but provides a compelling rationale for the incorporation of LUS as a complementary tool to reveal early congestion phenotypes that traditional assessment may miss. Taken together with studies demonstrating the prognostic significance of pulmonary congestion in dialysis, including its association with adverse cardiovascular outcomes [7,18], lung ultrasound should therefore be regarded not as a stand-alone therapeutic intervention but as a physiologically informed adjunct that refines the assessment of volume and hemodynamic status in routine hemodialysis care.

Although no study has shown that stethoscope auscultation improves mortality, it continues to serve as an essential bedside diagnostic aid. PoCUS should be seen in the same spirit, as the next step in the evolution of the physical examination, rather than being held to unrealistic, outcome-driven standards [39]. Integrating LUS into a multiparametric evaluation that combines auscultation, inspection, and bioimpedance improves the sensitivity and reproducibility of congestion assessment, allowing clinicians to identify subclinical pulmonary congestion that traditional methods may overlook.

### 3.2. Inferior Vena Cava (IVC) Ultrasound

The inferior vena cava (IVC) offers a window into right-sided filling pressures. Its diameter and respiratory variation, measured in the subxiphoid long-axis view, provide a noninvasive estimate of right atrial pressure (RAP) [12]. A plethoric IVC (>2.1 cm) with minimal inspiratory collapse suggests elevated RAP, whereas a narrow, highly collapsible IVC reflects lower RAP.

In dialysis patients, interpretation must remain context-specific. IVC dynamics primarily represent venous compliance and RAP, not absolute intravascular volume [14,40]. Changes observed during ultrafiltration may offer supportive information about hemodynamic behavior but should not be interpreted in isolation. Factors such as mechanical ventilation, elevated intra-abdominal pressure, and tricuspid regurgitation can influence IVC findings independently of actual volume status or congestion. Within a multiparametric PoCUS framework, combining LUS and venous Doppler evaluation, the IVC becomes a complementary rather than a stand-alone parameter. Although the inferior vena cava (IVC) is useful in cardiology to estimate right atrial pressure, it should not be interpreted in isolation as a marker of intravascular volume. Its clinical value in dialysis care lies primarily in understanding whether an increase in right atrial pressure is transmitted retrogradely to other venous territories. This concept of venous hypertension becomes physiologically meaningful only when the IVC is integrated with additional venous Doppler windows, particularly the portal vein where pulsatility more reliably reflects the hemodynamic impact of elevated right-sided pressures. Therefore, the IVC contributes to clinical decision-making not by quantifying volume, but by helping determine whether right atrial pressure elevations generate downstream venous congestion within a multiparametric PoCUS assessment.

### 3.3. Portal Vein Doppler

Doppler interrogation of the portal vein extends the hemodynamic assessment to the splanchnic compartment. Under normal conditions, portal flow is continuous and monophasic, buffered by hepatic sinusoids that dampen RAP oscillations. As venous congestion increases, these oscillations propagate to the portal system, resulting in pulsatile flow proportional to the degree of venous hypertension [9,14].

The ACUVEX study (Venous Excess in Hemodialysis) validated this approach, showing that portal vein pulsatility closely correlates with RAP and decreases dynamically during ultrafiltration. Patients with high pre-dialysis portal pulsatility experienced more intradialytic hypotension and lower tolerance to decongestion, supporting its role as a dynamic and prognostically relevant marker of venous hypertension [16].

### 3.4. Hepatic and Intrarenal Vein Doppler

Although the hepatic and intrarenal veins are included in the original VExUS score, they are not routinely assessed in dialysis practice. Accurate interpretation of hepatic venous flow requires ECG synchronization to differentiate systolic unreliable in arrhythmias [14]. Likewise, intrarenal Doppler assessment is technically challenging, as the kidneys are frequently atrophic and fibrotic, providing poor acoustic windows and inconsistent venous signals [14]. Consequently, these measurements offer limited feasibility and diagnostic yield in the hemodialysis population.

### 3.5. The Extended VExUS Concept

To improve feasibility, an extended VExUS framework incorporates superficial venous windows such as the internal jugular (IJV) and common femoral veins (CFV). The IJV, assessed at a 30–45° head elevation, aids in estimation of RAP based on parameters such as respiratory variation, the height of the venous collapse point, and changes in diameter or cross-sectional area during the Valsalva maneuver [41,42]. The CFV Doppler wave form also reflects distal transmisión of the RAP: the presence of systolic flow reduction or reversal indicates significant venous hypertension [43].

This extended approach enhances the clinical applicability of venous ultrasound in dialysis, where abdominal imaging may be limited by body habitus or position. By integrating pulmonary, caval, portal, and peripheral venous findings, clinicians obtain a comprehensive, physiology-based view of systemic congestion, supporting safer and more individualized ultrafiltration strategies. It is important to note that the diagnostic performance of each modality varies, and their clinical usefulness is maximized only when interpreted together within a multiparametric framework rather than in isolation.

### 3.6. Nutritional Ultrasound: Linking Muscle Health and Congestion

Beyond hemodynamic assessment, ultrasound also enables the evaluation of nutritional and functional status. Nutritional ultrasound (NUS) is a standardized bedside technique that quantifies muscle morphology and adipose tissue distribution using reproducible measurements such as quadriceps thickness, cross-sectional area, and echogenicity—objective indicators of protein-energy wasting (PEW) and inflammation [24].

In the study by De La Flor et al. involving 74 patients on maintenance HD, NUS of the quadriceps rectus femoris and preperitoneal visceral fat correlated strongly with appendicular skeletal muscle mass, handgrip strength, and phase angle derived from bioimpedance. The latter provides complementary information on body composition, allowing distiction between lean tissue and fat tissue mass. The prevalence of confirmed and severe sarcopenia reached 40% and 20%, respectively. Ultrasound-derived muscle parameters such as anterior–posterior quadriceps thickness ≤ 8 mm or muscle area indexed to height ≤ 0.9 cm^2^/m^2^, showed good diagnostic performance and remained independent of hydration status, underscoring their value in dialysis populations [25]. Additional evidence supporting the role of muscle ultrasound in hemodialysis comes from the study by Güner et al. [44] , which evaluated 45 maintenance hemodialysis patients and demonstrated that rectus femoris cross-sectional area (RFCSA) was independently associated with both sarcopenia and malnutrition. RFCSA and muscle thickness parameters showed meaningful discrimination of nutritional status, reinforcing the potential diagnostic value of muscle ultrasonography in this setting. However, despite these promising findings, the current evidence base remains limited to small, single-center studies, and larger multicenter investigations are needed to validate cut-off points, improve reproducibility, and determine the prognostic relevance of NUS in routine dialysis practice. Although these findings are promising, the current evidence base for NUS in dialysis remains limited and derives mostly from small, single-center studies. Larger, multicenter investigations are needed to validate these measurements, establish reference values, and determine their prognostic relevance in routine hemodialysis practice.

**Figure 2:**
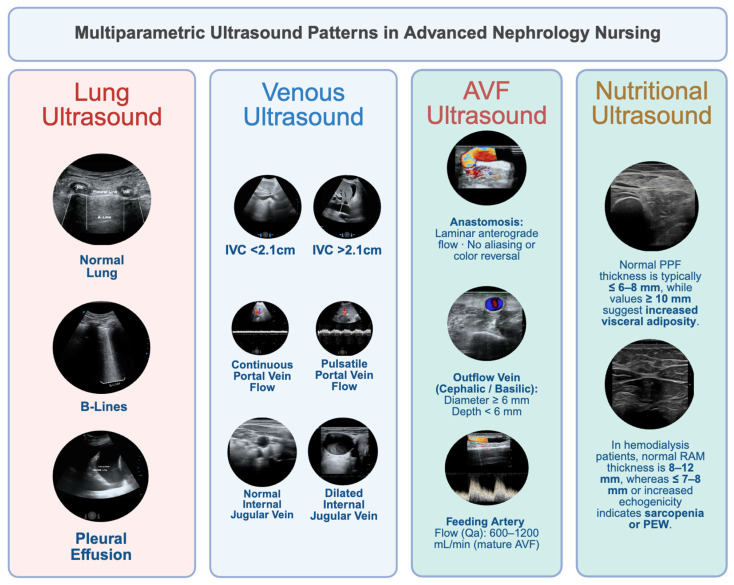
Multiparametric Ultrasound Patterns in Advanced Nephrology Nursing. This figure summarizes key PoCUS patterns used by advanced practice nurses across four domains. Lung ultrasound (LUS) shows normal lung sliding, B-lines indicating interstitial congestion, and pleural effusion. Venous ultrasound includes IVC assessment (<2.1 cm vs. >2.1 cm), portal vein Doppler patterns (continuous vs. pulsatile flow), and normal versus dilated internal jugular vein (IJV). AVF ultrasound demonstrates laminar anastomotic flow, adequate outflow-vein characteristics (diameter ≥ 6 mm; depth < 6 mm), and typical feeding-artery flow (Qa 600–1200 mL/min). Nutritional ultrasound (NUS) illustrates normal preperitoneal fat (PPF ≤ 6–8 mm), increased visceral adiposity (PPF ≥ 10 mm), and rectus abdominis muscle (RAM) morphology, where reduced thickness (≤7–8 mm) or increased echogenicity suggests sarcopenia or protein-energy wasting (PEW). Abbreviations: AVF = arteriovenous fistula; IVC = inferior vena cava; IJV = internal jugular vein; LUS = lung ultrasound; NUS = nutritional ultrasound; PPF = preperitoneal fat; PEW = protein-energy wasting; RAM = rectus abdominis muscle; Qa = access blood flow.

These findings highlight the interdependence of malnutrition and congestion. Chronic venous hypertension and recurrent volume overload promote inflammation and catabolism, while sarcopenia and reduced muscle quality diminish venous capacitance and ultrafiltration tolerance. Integrating NUS within the same multiparametric PoCUS framework that includes pulmonary, IVC, and portal assessments allows a more complete characterization of the patient—linking structural (muscular), metabolic (nutritional), and hemodynamic (congestive) domains (Figure 2).

Ultimately, NUS broadens the concept of multiparametric ultrasound evaluation, integrating muscle structure, nutritional reserve and body composition into the same physiological continuum as pulmonary and venous congestion. This unified perspective supports a more holistic, patient-centered approach to precision care in dialysis.

## 4. Defining the Role of Advanced Practice Nurses in PoCUS-Enhanced Nephrology Care

The integration of PoCUS into nephrology nursing requires a clear definition of the professional profile responsible for image acquisition and preliminary interpretation. In this model, examinations are performed by APNs or by registered nurses who have completed structured, competency-based ultrasound training. According to the International Council of Nurses [45,46], an APN is a registered nurse with expert knowledge, advanced clinical reasoning, and expanded decision-making skills acquired through graduate-level education and formal clinical supervision. Although regulatory pathways differ globally, their conceptual alignment is consistent. In the United States, advanced practice providers, including nurse practitioners and physician assistants perform diagnostic and therapeutic tasks under collaborative agreements with physicians, functioning as mid-level clinicians with independent or semi-independent scopes of practice [46].

In European and Latin American health systems, where this model was developed, registered nurses may acquire advanced diagnostic competencies such as PoCUS through accredited programs, supervised hands-on training, and competency validation [47]. Within this collaborative structure, APNs do not replace medical evaluation but act as highly trained clinical partners who integrate ultrasound findings with hemodynamic, analytical, and symptomatic data. Their role centers on early identification of physiological instability, preliminary interpretation of congestion or functional decline, and timely communication with the nephrologist to support individualized therapeutic decisions.

## 5. Integration of PoCUS into Advanced Nephrology Nursing Practice

The incorporation of point-of-care ultrasonography (PoCUS) into nephrology nursing marks a decisive step toward physiology-based, patient-centered care.

Traditionally viewed as a physician-led diagnostic domain, ultrasound is increasingly becoming an essential skill for APNs, broadening their role to encompass image interpretation and clinical integration as key members of the healthcare team [48]. By integrating real-time hemodynamic and nutritional information at the bedside, APNs are uniquely positioned to anticipate instability, guide individualized ultrafiltration, and collaborate with physicians to optimize treatment. This paradigm shift redefines nursing within the framework of precision medicine. Through the simultaneous assessment of pulmonary, venous, vascular-access, and muscular domains, nurses can construct a multiparametric physiological profile for each patient, bridging clinical findings, biosensor data, and laboratory trends into a single interpretative continuum (Table 1).

In this model, the dialysis unit evolves into a diagnostic environment where congestion, nutritional decline, and vascular access status are visualized and managed proactively, fostering a new level of interdisciplinary communication between nursing and medical staff.

PoCUS thus represents not merely a tool, but a diagnostic extension of advanced nursing reasoning. It supports early recognition of congestion, functional deterioration, and analytical imbalance, allowing timely alerts and collaborative therapeutic adjustments that reinforce safety and patient outcomes [55].

## 6. Clinical Protocol: Nursing-Led PoCUS Workflow

To enhance clarity and reproducibility, the proposed PoCUS workflow has been reformatted into a cyclic diagram that illustrates its continuous application across the predialysis, intradialytic, and postdialysis phases. This representation highlights the iterative nature of physiologic reassessment and explicitly incorporates the points at which multidisciplinary consultation particularly with nephrologists, cardiologists, and vascular access specialists, enhances diagnostic accuracy and mitigates clinical risk.

The nursing-led PoCUS workflow adopts a structured approach that integrates clinical examination, sonographic findings, and, when available, dialysis biosensor data. This model enables APNs to identify early congestion, assess ultrafiltration tolerance, and provide targeted input to the nephrologist, consistent with emerging evidence supporting nurse-performed ultrasound in nephrology practice. Below is the protocol we recommend.

**Step 1—Initial Assessment**

At patient arrival, pre-dialysis weight, blood pressure, heart rate, and symptoms (dyspnea, dizziness, edema) are recorded. The current weight is compared with the prescribed dry weight to calculate interdialytic weight gain (IWG).

- IWG < 1% or presence of hypotension/dizziness suggests hypovolemia and a high risk of UF intolerance.
- IWG > 3–4% or presence of dyspnea or edema indicates suspected congestion.

This initial screening defines whether the patient requires a conservative approach or a focused ultrasound examination prior to connection.

**Step 2—First Decision**

- **Non-congestive phenotype:** When no B-lines are detected on LUS and the IVC shows normal diameter and collapsibility, a conservative UF strategy or temporary UF pause is advised. The nurse continues close clinical and hemodynamic monitoring.
- **Suspected congestion:** If clinical findings suggest fluid overload, the nurse proceeds to focused PoCUS evaluation before initiating dialysis, as recommended in studies validating LUS and IVC assessment in hemodialysis care

**Step 3—Bedside PoCUS Screening**

The targeted ultrasound exam includes the LUS, IVC, and portal vein (PV) Doppler, together with arteriovenous fistula (AVF) assessment and NUS where applicable. Findings define the predominant hemodynamic and functional phenotype [9]:

- **No congestion:** Maintain UF target and schedule next evaluation; consider cardiac PoCUS if diagnostic uncertainty persists.
- **Predominant tissue congestion:** Initiate a controlled decongestive strategy with gradual dry-weight reduction and biosensor-guided UF. If dyspnea persists, consider extended UF guided by biosensor feedback and refer for cardiac PoCUS to assess left-sided pressures.
- **Predominant venous congestion:** Use progressive decompression rather than aggressive UF; refer for cardiac PoCUS to evaluate right ventricular function.
- **Mixed congestion:** Implement a stepwise UF approach, targeting UF < 10 mL/kg/h, reducing dry weight by 0.3–0.5 kg per session, and re-evaluating after 2–3 sessions. Refer for Cardiac PoCUS may help determine the dominant mechanism of congestion.

**Figure 3:**
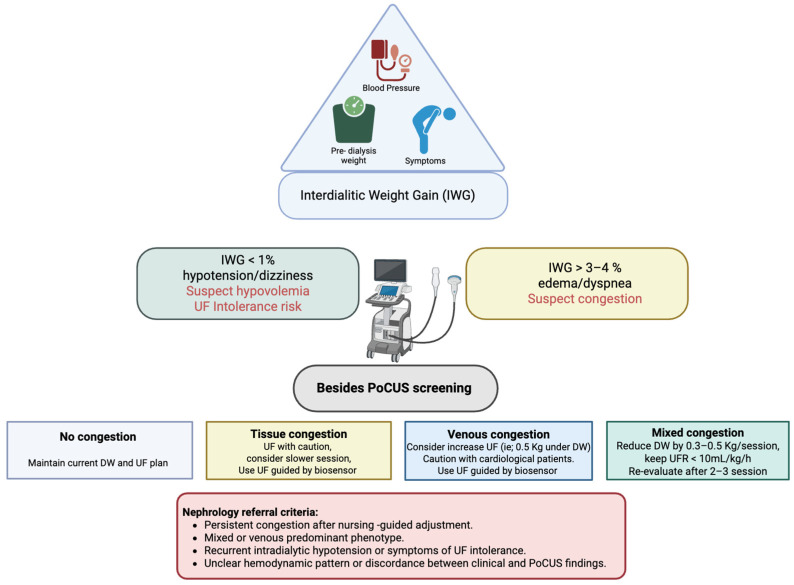
Clinical Integration of PoCUS in the Nursing Phase of Hemodialysis. Schematic representation of the nursing-led workflow integrating PoCUS into pre-dialysis assessment and ultrafiltration (UF) decision-making. At each step of this workflow, APN-led findings are interpreted within a multidisciplinary framework. Abnormal pulmonary congestion patterns, venous Doppler abnormalities, or vascular-access concerns prompt targeted consultation with nephrologists, cardiologists, or access specialists, ensuring that PoCUS findings complement rather than replace expert clinical judgment. This collaborative approach reduces the risk of misinterpretation and supports safer therapeutic decision-making. Abbreviations: PoCUS, point-of-care ultrasound; UF, ultrafiltration; IWG, interdialytic weight gain; DW, dry weight.

Nutritional ultrasound (quadriceps and preperitoneal fat) is performed periodically or when functional decline is suspected to identify sarcopenia or protein-energy wasting (PEW). Vascular access ultrasound, pre-cannulation mapping, real-time guidance, and Doppler surveillance ensures safety, minimizes infiltration, and preserves long-term patency (Figure 3).

## 7. Implementation of the Nursing-Led PoCUS Program: Our Experience

Following the design of the protocol, a structured implementation plan was developed to ensure uniform adoption across the dialysis unit. The process began with a targeted educational phase in which three reference nurses, two from the HD unit and one from the peritoneal dialysis team, received dedicated training in lung, vascular, and nutritional ultrasound.

After this period of supervised instruction, these reference nurses delivered theoretical and hands-on sessions to the rest of the team, promoting peer learning and skill dissemination. Regular bedside mentoring during routine clinical practice reinforced image acquisition, interpretation accuracy, and confidence in the use of ultrasound-guided assessment.

Once competency was achieved, the PoCUS protocol was systematically integrated into the daily workflow. Each patient underwent a baseline ultrasound upon enrollment in the program, followed by a reassessment after one month and subsequently on a monthly basis, coinciding with laboratory evaluations. Additional scans were performed whenever signs or symptoms of congestion were present or during hospitalization. In hemodialysis, the scan was ideally performed immediately before connection, enabling individualized adjustment of the UF target and dry weight. In peritoneal dialysis, PoCUS was incorporated at program entry and during adequacy evaluations or whenever congestion was suspected. To support safe and reproducible implementation, training in PoCUS for advanced practice nurses should follow a structured, competency-based framework. Standard operating procedures (SOPs) for each ultrasound domain lung, IVC, portal vein, vascular-access, and muscle assessment, should include clear specifications for probe selection, patient positioning, scanning planes, image optimization, and minimum documentation standards. These SOPs promote consistency across operators and enable reproducible longitudinal assessments.

Training delivery typically integrates didactic instruction with supervised hands-on scanning and case-based interpretation. Recent proposals calling for the standardization of PoCUS training in nephrology emphasize the importance of a minimum number of supervised examinations per domain, systematic exposure to normal and pathological patterns, and formal evaluation of image acquisition and interpretative competency [55,56]. Competency maintenance requires ongoing practice, periodic image review, and structured feedback loops within multidisciplinary PoCUS teams, ensuring that APN-led examinations remain physiologically grounded and clinically safe.

In addition, the development of shared training resources such as annotated image libraries, repositories of pathological findings, and checklists for common scanning pitfalls further enhances reproducibility and facilitates adoption across dialysis units. Aligning training with emerging PoCUS frameworks ensures that implementation is standardized, auditable, and adaptable to diverse clinical environments, supporting the long-term integration of PoCUS into advanced nursing practice in nephrology [55,56].

## 8. Operational and Clinical Integration

PoCUS is applied cyclically and reproducibly across care phases:

- **Baseline assessment:** at program initiation to establish individual hemodynamic and nutritional profiles.
- **Monthly reassessment:** coinciding with laboratory and clinical reviews.
- **Event-triggered evaluation:** during episodes of clinical congestion, hospitalization, or UF intolerance.

In HD, PoCUS is ideally performed at connection to the circuit, allowing real-time UF prescription tailoring. In peritoneal dialysis (PD), ultrasound is synchronized with adequacy visits to maintain continuity across modalities. Although the core workflow is shared across dialysis modalities (Figure 3), specific adaptations are required in HD and PD. In HD, intradialytic reassessment focuses on the dynamic effects of ultrafiltration on pulmonary and venous congestion, whereas in PD the evaluation emphasizes chronic fluid distribution. These modality-specific considerations have been integrated into the updated workflow diagram to reflect their practical relevance.

Portable ultrasound systems enable chairside evaluation, transforming the dialysis unit into a point-of-care physiology laboratory where decisions are individualized and continuously refined. This structured integration ensures continuity of data, reinforces clinical reasoning, and enhances safety through early detection of instability and informed therapeutic adjustment. In addition, recent developments in artificial intelligence (AI) are beginning to enhance the functionality of portable ultrasound systems. As highlighted in current PoCUS frameworks, modern handheld platforms increasingly incorporate algorithms that assist with automatic plane acquisition, real-time labeling of anatomical structures, and semiautomated quantification of cardiac or vascular parameters [34,37]. These tools initially developed to support standardized echocardiographic measurements such as chamber dimensions or left ventricular ejection fraction, have the potential to reduce operator dependency and improve consistency in image acquisition. Importantly, the progressive incorporation of AI-based guidance aligns with ongoing efforts to standardize PoCUS training and competency assessment in nephrology, where minimizing inter-operator variability is essential for safe and reproducible implementation [56]. Although their use in dialysis care is still emerging, AI-assisted acquisition may further facilitate the adoption of PoCUS by advanced practice nurses and help standardize complex measurements in routine workflows. The operational integration of PoCUS also carries inherent clinical risks that must be acknowledged to ensure safe implementation. Misinterpretation of sonographic findings—particularly when images are obtained suboptimally or interpreted outside the appropriate physiologic context—may lead to incorrect assumptions about volume status, cardiac filling pressures, or venous congestion. Common pitfalls include overreliance on single parameters (such as IVC diameter), failure to recognize technical artifacts, or attributing Doppler abnormalities to congestion when they may reflect ventilation patterns, arrhythmias, or unfavorable insonation angles [12,40]. These limitations underscore the necessity of a multiparametric, physiology-based approach and of integrating PoCUS within multidisciplinary decision-making rather than using it as an isolated diagnostic tool.

Operational challenges also arise from the need to maintain image quality standards, ensure regular equipment calibration, and prevent “scope creep,” in which PoCUS is used beyond the operator’s formal training or competency. Structured feedback mechanisms, periodic review of image portfolios, and collaboration with nephrologists, cardiologists, and radiologists help mitigate these risks and reinforce the safe application of PoCUS findings in dialysis care. Embedding these safeguards into routine workflows is essential to maintain diagnostic integrity and protect patient safety.

## 9. Toward Precision Nursing

The integration of PoCUS into nephrology nursing practice represents a shift from procedural assistance to active diagnostic participation [56].

By uniting the assessment of congestion, nutrition, and vascular access within a single interpretative framework, APNs become central agents of precision medicine, able to identify physiological patterns, anticipate instability, and personalize treatment in real time. This model transforms the dialysis unit into a dynamic diagnostic ecosystem, where medical and nursing expertise converge through shared reasoning, continuous feedback, and real-time data interpretation.

Beyond congestion, advanced nephrology nurses act as guardians of physiological equilibrium [49]. They monitor and interpret key parameters that define the cardiorenal continuum like anemia, mineral bone disorder, blood pressure control, potassium balance, and volume status, detecting early deviations before clinical deterioration occurs. Through this vigilant observation, they alert the nephrologist to evolving trends that may require therapeutic action: adjustment of erythropoiesis-stimulating therapy, optimization of iron supplementation, modification of phosphate binders or vitamin D analogs, adaptation of antihypertensive strategies, or refinement of dialysis prescriptions to improve ultrafiltration tolerance and metabolic control.

This role as clinical sentinels integrates analytical, physiological, and sonographic information into a coherent and proactive management process. By translating complex parameters into actionable insights, nurses not only accompany but also complement the physician’s decision-making, ensuring that early physiological changes lead to precise therapeutic interventions.

Education, structured training, and competency validation remain the foundation of this model. Standardized curricula, supervised practice, and interobserver calibration guarantee that nursing-performed PoCUS and NUS achieve diagnostic validity comparable to physician-led assessments, reinforcing safety and clinical credibility across teams [50,51]. Ultimately, precision nursing encapsulates the essence of this transformation: the convergence of physiology, clinical reasoning, and technology in the hands of nursing professionals. In doing so, they embody the future of nephrology: a discipline where diagnostic insight, therapeutic precision, and patient partnership merge into a single, continuous process of care.

Building upon the experience of critical care nursing, the implementation of PoCUS in advanced nephrology practice should rely on clearly defined, competency-based standards. Based on existing ICU nursing models, we consider a reasonable baseline to include: (1) a structured introductory course with supervised hands-on training, (2) ≥30 supervised examinations for each core domain, namely lung ultrasound (identification of B-lines, pleural patterns, and aeration changes) and venous congestion assessment using IVC evaluation and simplified Doppler-based profiles appropriate to the nursing scope, and (3) periodic competency verification through structured image-review sessions and semi-annual external audits to prevent the well-known risk of overestimating ability after short courses [52]. These training thresholds align with emerging international frameworks such as the IAPN model and national efforts to standardize PoCUS curricula in nephrology [50,51]. Importantly, following the precedent of critical care nursing, we do not consider PoCUS an additional time-consuming task: focused lung and venous congestion assessments can be seamlessly integrated into the standard initial evaluation of patients with acute kidney injury, fluid overload, or cardiorenal syndromes, replacing rather than adding to less informative elements of the physical examination. Regarding reimbursement, international experience shows that nurse-performed PoCUS is rarely billed as an independent procedure; instead, healthcare systems incorporate it into expanded scopes of practice supported through credentialing pathways, institutional recognition, and protected training time. We therefore advocate for a similar model in nephrology, where PoCUS is formally recognized, structurally supported, and competency-verified—rather than reimbursed per scan. The implementation of an APN-led PoCUS model should also be framed within a broader interdisciplinary structure. Although nurses may acquire and integrate point-of-care findings at the bedside, interpretation and subsequent therapeutic decisions frequently require collaboration with nephrologists, cardiologists, radiologists, and vascular-access specialists. This interdisciplinary exchange ensures that PoCUS findings are contextualized appropriately, reduces the risk of diagnostic overconfidence, and maintains alignment with established standards of care.

From an operational perspective, the adoption of PoCUS entails direct and indirect costs, including equipment acquisition, device maintenance, protected time for training, and periodic competency reassessment. Maintaining proficiency requires continued practice, image portfolio review, and participation in structured feedback processes, consistent with current recommendations for competency-based PoCUS training in nephrology [55,56]. These ongoing requirements underscore that PoCUS is not a static skill, but one that must be continuously reinforced to sustain diagnostic accuracy and patient safety.

In some settings, alternative models such as the incorporation of specialized sonographers within dialysis units, may provide complementary or substitute approaches for delivering ultrasound-based diagnostics. The choice between APN-led and sonographer-led models depends on institutional resources, workforce structure, and the desired level of point-of-care integration. Importantly, reimbursement pathways vary considerably across health systems and often recognize ultrasound services only when performed or supervised by credentialed providers. Where applicable, existing policies require appropriate documentation, credentialing, and linkage to medical decision-making, and these constraints should be taken into account when designing local implementation strategies.

Overall, APN-led PoCUS should be viewed not as a standalone diagnostic service but as a structured, collaborative, and resource-dependent process that contributes to individualized care when implemented within a multidisciplinary and quality-controlled framework.

## 10. Conclusions

The integration of PoCUS into nephrology nursing transforms the discipline from task-oriented to diagnostic-driven practice. By uniting hemodynamic, metabolic, and structural insights, nurses become the physiological sentinels of the cardiorenal continuum, detecting early congestion, guiding ultrafiltration, preserving vascular access, and safeguarding nutritional integrity. This approach redefines the boundaries of collaboration between nursing and nephrology, setting the foundation for precision nursing: a model where evidence, physiology, and empathy converge to achieve safer and more personalized renal replacement therapy.

## Figures and Tables

**Table 1:** Nurse-performed point-of-care ultrasound (PoCUS) studies across clinical settings.

No.	Clinical Area	Authors (Year); Journal	Design/*N*	Technique	Operator	Main Findings	Limitations
1	Hemodialysis	Lee et al. [49]	Pragmatic intervention, *n* = 13 HD patients	8-zone LUS	Nurses	Nurse-led LUS-guided dry-weight titration was feasible and safe, showing a trend toward lower ambulatory blood pressure.	Small sample size (*n* = 13) and single-center design; short follow-up; surrogate outcomes only; limited generalizability.
2	Critical care (mixed ICU)	Smits et al. [50]	Prospective observational	Thoracic PoCUS (LUS + pleura)	ICU nurses (“UltraNurses”)	Nurse-performed PoCUS led to changes in clinical management in >25% of cases, mainly affecting fluid decisions within 8 h.	Observational study without control group; heterogeneous ICU population; findings may not extrapolate to chronic dialysis settings.
3	Heart failure (outpatient)	Gundersen et al. [51]	Randomised controlled trial, HF clinic	LUS + IVC	Heart-failure nurses	Nurse-performed ultrasound improved volume assessment accuracy and altered management compared with standard evaluation.	Single-clinic setting; specialized HF nurses may not reflect training profiles of dialysis staff; potential learning-curve effects.
4	Heart failure (outpatient)	Dalen et al. [52]	Observational, HF clinic	LUS + IVC	Nurses	High feasibility and good agreement with reference sonographers for pleural effusion and IVC evaluation after short training.	Small sample and observational design; moderate inter-operator variability; no long-term clinical outcomes measured.
5	Hemodialysis vascular access	Hill K et al. [53]	Pre-test/post-test training study, *n* = 15 nurses + 17 patients	AVF assessment + cannula placement	HD nurses	After PoCUS education, nurses reported increased confidence in cannulation; patients supported PoCUS use and it potentially avoided transfers due to cannulation difficulties.	Pre/post design without randomization; small cohort; subjective nurse-reported outcomes; lacks procedural performance metrics.
6	Hemodialysis vascular access	Grosu I et al. [54]	Survey, outpatient HD unit	AVF PoCUS assessment	HD nursing staff	Nurses viewed AVF-PoCUS as a useful tool; positive attitude toward implementation, though confidence in cannulation decreased slightly over 5 years.	Survey-based methodology; self-reported confidence prone to bias; absence of objective ultrasound performance data.

This table summarizes representative studies evaluating the implementation and outcomes of nurse-performed PoCUS in hemodialysis, cardiology, and critical care. The evidence demonstrates the feasibility, safety, and diagnostic reliability of lung ultrasound (LUS) and venous Doppler techniques performed by trained nurses or nurse practitioners, as well as the growing application of vascular access ultrasound in dialysis. Studies in hemodialysis confirm that nurse-led LUS-guided dry-weight titration improves blood pressure control, while vascular access ultrasound enhances cannulation safety and nurse confidence. To provide a balanced interpretation of the available evidence, Table 1 includes a dedicated column summarizing the main methodological limitations of each study. Most investigations were small and single-center, frequently observational in design, and therefore susceptible to selection bias, operator-dependent variability, and limited generalizability. These constraints should be taken into account when interpreting the overall role of nurse-performed PoCUS in nephrology practice. Abbreviations: AVF = Arteriovenous fistula; HD = Hemodialysis; IVC = Inferior vena cava; LUS = Lung ultrasound; PoCUS = Point-of-care ultrasonography.

## Data Availability

No new data were created or analyzed in this study. Data sharing is not applicable to this article.
